# Incidence of *De Novo* Post-Transplant Malignancies in Thai Adult Kidney Transplant Recipients: A Single-Center, Population-Controlled, Retrospective Cohort Study at the Highest Volume Kidney Transplant Center in Thailand

**DOI:** 10.3389/ti.2024.11614

**Published:** 2024-02-26

**Authors:** Praopilad Srisuwarn, Napun Sutharattanapong, Sinee Disthabanchong, Surasak Kantachuvesiri, Chagriya Kitiyakara, Bunyong Phakdeekitcharoen, Atiporn Ingsathit, Vasant Sumethkul

**Affiliations:** ^1^ Department of Medicine, Faculty of Medicine, Ramathibodi Hospital, Mahidol University, Bangkok, Thailand; ^2^ Division of Nephrology, Department of Medicine, Faculty of Medicine, Ramathibodi Hospital, Mahidol University, Bangkok, Thailand; ^3^ Excellence Center for Organ Transplantation, Faculty of Medicine, Ramathibodi Hospital, Mahidol University, Bangkok, Thailand; ^4^ Department of Clinical Epidemiology and Biostatistics, Faculty of Medicine, Ramathibodi Hospital, Mahidol University, Bangkok, Thailand

**Keywords:** kidney transplantation, cancer, adult, incidence, Thailand

## Abstract

Kidney transplant recipients (KTRs) are at increased risk of developing *de novo* post-transplant malignancies (PTMs), with regional differences in types with excess risk compared to the general population. A single-center, population-controlled, retrospective cohort study was conducted at a tertiary care center in Thailand among all adults who underwent their first kidney transplant from 1986 to 2018. Standardized incidence ratios (SIRs) of malignancy by age, sex, and place of residence were obtained using data from the National Cancer Registry of Thailand as population control. There were 2,024 KTRs [mean age, 42.4 years (SD 11.4); female patients, 38.6%] during 16,495 person-years at risk. Of these, 125 patients (6.2%) developed 133 *de novo* PTMs. The SIR for all PTMs was 3.85 (95% CI 3.22, 4.56), and for pooled solid and hematologic PTMs, it was 3.32 (95% CI 2.73, 3.99). Urothelial malignancies had the largest excess risk, especially in women [female SIR 114.7 (95% CI 66.8, 183.6); male SIR 17.5 (95% CI 8.72, 31.2)]. The next two most common cancers were non-Hodgkin’s lymphoma and skin cancer [SIR 20.3 (95% CI 13.6, 29.1) and 24.7 (95% CI 15.3-37.8), respectively]. Future studies are needed to identify the risk factors and assess the need for systematic screening among PTMs with excess risk in KTRs.

## Graphical Abstract

**Figure:**
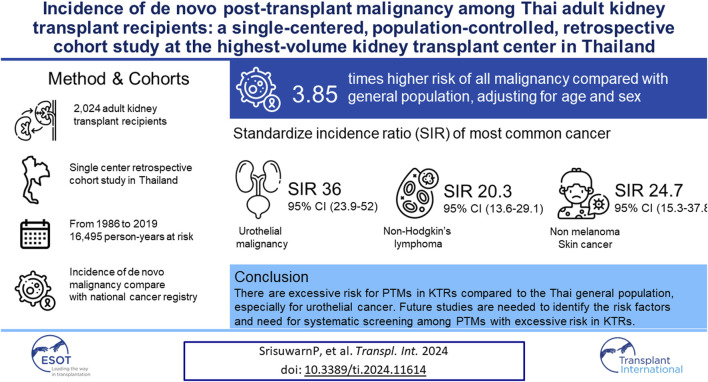

## Introduction

Post-transplant malignancy (PTM) is one of the most devastating long-term complications for kidney transplant recipients (KTRs), leading to death with a functioning graft [1]. The excess risk of PTMs in KTRs is 2.9–3.9 times that of the general population. The risk is even higher for virus-related cancers, such as Kaposi’s sarcoma, post-transplant lymphoproliferative disorder, and non-melanoma skin cancer [2–7]. The excess risk is mainly due to immunosuppressive agents that increase vulnerability to oncogenic viral infection, disrupt the ability of the immune surveillance systems to detect and remove abnormal cells, and interfere with cell repair mechanisms [8]. However, the risk of each cancer may vary by geographical area due to site-specific environmental exposures and genetics.

Therefore, the incidence of PTMs among KTRs in each region must be estimated to allow policymakers to plan for appropriate service provision. To date, no study has estimated the excess risk of PTMs in Thai KTRs compared with the Thai general population. Thus, we aimed to estimate the incidence of PTMs in adult Thai KTRs at the highest-volume transplant center in Thailand.

## Patients and Methods

### Study Design, Patients, and Setting

This was a single-center, retrospective cohort study conducted at Ramathibodi Hospital, a tertiary care university hospital that performs the highest volume of kidney transplants in the country, accounting for one-quarter of the cumulative kidney transplant surgeries in Thailand. This study included consecutive adult patients aged ≥18 years who received a first kidney transplant between 1 January 1986 and 30 July 2018 and had ≥1 month of follow-up. All recipients were followed up from the date of kidney transplantation to the date of a diagnosis of *de novo* incident cancer, all-cause death, graft failure, loss to follow-up, or administrative censoring on 31 December 2019, whichever occurred first. The exclusion criteria were cancer occurring after graft failure and cancer occurring before or ≤1 month following transplantation. The study was approved by the Institutional Review Board, Faculty of Medicine, Ramathibodi Hospital (MURA2022/503). Informed consent was not required due to the de-identification of patient data. This study was conducted in accordance with the tenets of the Declaration of Helsinki.

### Data Collection

Data were collected from three sources. First, incidence data for the Thai general population were obtained from the Thai National Cancer Institute’s (NCI) nationwide cancer data registry. We chose to compare incident malignancy rates between our KTRs and the Thai general population between 2013 and 2015. We only selected the 2013–2015 period because the place of residence to be matched with that of our KTRs in the registry had been validated by cross-checking with the Ministry of Interior’s National Civil Registration records [9]. Additionally, data after 2016 were not available when we conducted the analysis. Moreover, the national coverage of the Thai NCI has gradually grown since the establishment of the first population-based only on data from Chiangm Mai province in 1986 [10]. Thus, adjusting for calendar year using historical subnational coverage would have reduced our sample size and power to detect any significant results. Diagnoses were initially coded by registry personnel using the International Classification of Diseases for Oncology (ICD-O, third edition), and the coding demonstrated good reliability [9]. These codes were then transformed to the International Classification of Diseases, Tenth Revision (ICD 10) for analysis. Second, we collected baseline patient data from the Ramathibodi KTR database, including age, sex, region of residence, and type and date of transplantation. Third, we manually reviewed hospital electronic medical records to collect outcomes and the date of outcomes. Diagnoses of *de novo* PTMs were ascertained by histopathologic confirmation or compatible radiographic studies with serologic testing. These were then categorized according to the ICD-10.

### Immunosuppressive Regimens

Immunosuppressive regimens for induction and maintenance therapy in KTRs were carefully selected based on the immunologic risks of the patients. Induction regimens included methylprednisolone with or without an interleukin-2 receptor antagonist or anti-thyroglobulin. The maintenance medications were mainly calcineurin inhibitor (CNI)-based regimens. A typical combination of these regimens before 2000 was cyclosporine-azathioprine-prednisolone, which gradually changed to tacrolimus-mycophenolate mofetil-prednisolone. Other regimens were used much less frequently, and they included CNI-mTOR inhibitor-prednisolone and CNI-prednisolone.

### Cancer Screening and Surveillance in Kidney Transplant Candidates and Recipients

At our center, age-appropriate pre-transplant cancer screening and abdominal ultrasound have been performed since January 2013. Post-transplant cancer surveillance was opportunistic and consistent with standard cancer screening recommended for the general population [11].

### Outcomes

The outcome was the standardized incidence ratio (SIR), which represents the excess risk of malignancy compared to the Thai general population.

### Statistical Analysis

Patient characteristics were analyzed descriptively. Continuous data are presented as mean (SD) or median (interquartile range), as appropriate. Categorical data are presented as frequencies (%).

The excess risk of *de novo* PTM in KTRs compared to the general population was estimated by SIR by indirect standardization of the Thai general population. Confidence intervals were estimated using the Poisson distribution. We selected the 2013–2015 data from the Thai NCI, which used the projected Thai population in mid-2014 based on the 2010 national population census. We defined the start of the time at risk as 1 month after the date of the kidney transplant to exclude non-*de novo* PTMs. We defined the end of the time at risk as incident *de novo* PTM, loss to follow-up, all-cause mortality, or administrative censoring at the end of the study period, whichever happened first. We also reported the incidence and SIRs stratified by sex, age at kidney transplant, duration of follow-up after kidney transplant, and transplant era of the top three cancers with the most excess risk. To avoid underestimating the incidence due to multiple *de novo* PTMs, if a second or third *de novo* PTM occurred, we generated time at risk from 1-month post-transplant to the date of the second or third incident PTM. Statistical analyses were performed using Stata 15.1 (StataCorp. 2017. College Station, TX). A non-null 95% confidence interval (CI) was considered significant.

## Results

### Characteristics of the Kidney Transplant Recipients

**FIGURE 1:**
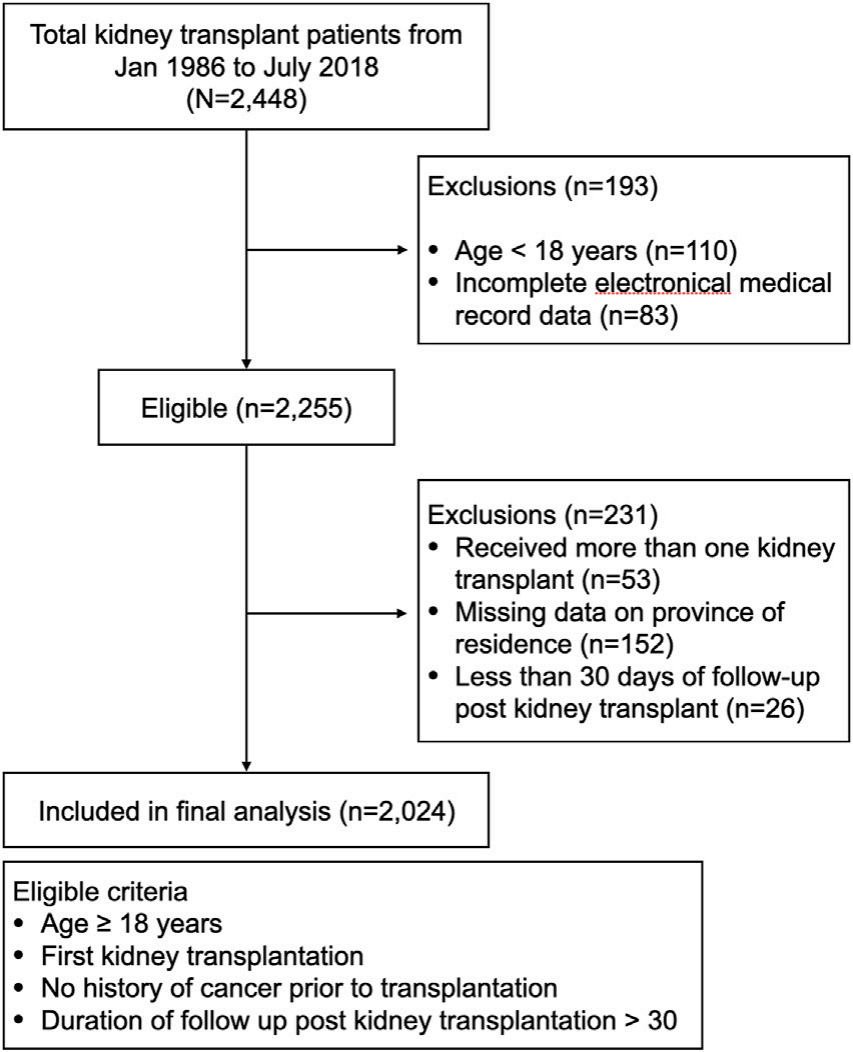
Flowchart of patients in the study.

During the 32-year period from January 1986 to July 2018, there were 2,448 kidney transplantations. After exclusions, a total of 2,024 cases were included in the final analysis, contributing to 16,495 patient-years of risk time in the follow-up (Figure 1; Table 1). The mean age at transplantation was 42.4 years (SD 11.4), and 38.6% of the subjects were women (Table 1). The median follow-up time was 6.4 years (interquartile range 3.21, 11.5) (Table 1).

**TABLE 1:** Characteristics of all kidney transplants.

Characteristic	All kidney transplants
Total number	2,024
Female patients, *n* (%)	781 (38.6)
Deceased donor	998 (49.3)
Age at transplantation, mean ± SD, y	42.4 ± 11.4
Male patients	42.6 ± 11.7
Female patients	42.2 ± 11.0
Region of residence in Thailand, *n* (%)	
Central	1,273 (63.1)
North-eastern	210 (10.4)
Northern	162 (8.0)
Eastern	220 (10.9)
Southern	151 (7.5)
Total person-time-at-risk, y	16,382
Male patients	10,127
Female patients	6,254
Year of transplantation, *n* (%)	
Before 1989	15 (0.7)
1989–1998	302 (14.9)
1999–2008	534 (26.4)
2009–2018	1,173 (58.0)
Length of follow-up in years, *n* (%)	
<1	82 (4.0)
1–4	706 (34.9)
5–9	618 (30.5)
10–14	313 (15.5)
15–19	178 (8.8)
≥20	127 (6.3)
Length of follow-up, median (IQR), y	6.35 (3.21, 11.5)
Male patients	6.39 (3.32, 11.6)
Female patients	6.19 (3.15, 11.4)

Abbreviations: IQR, interquartile range; SD, standard deviation.

### Incidence of *De Novo* Post-Transplant Malignancy

A total of 133 *de novo* PTMs were identified in 125 (6.2%) patients. In total, eight (6%) patients developed secondary cancer, including 2 non-melanoma skin cancers, 2 liver and biliary cancers, 1 prostate cancer, 1 kidney cancer, 1 urothelial cancer, and 1 colorectal cancer. The incidence by type of PTMS, age at diagnosis, and time from transplantation to diagnosis are shown in Table 2. The most common PTM was urothelial cancer (*n* = 28), accounting for one-fifth of all PTMs in the cohort, followed by non-Hodgkin’s lymphoma (NHL) at 22% and non-melanoma skin cancer at 16%.

**TABLE 2:** Distribution and clinical characteristics of malignancies following kidney transplantation by sex.

	Male patients	Female patients	All	Percentage	Age at transplantation, mean ± SD, y	Age at diagnosis of PTM, mean ± SD, y	Time from transplant to PTM, median (IQR), y
Solid							
Urothelial	11	17	28	35.0	49.7 ± 9.93	58.7 ± 12.7	6.63 (4.55, 10.4)
Prostate	9		9	11.3	56.3 ± 7.22	68.8 ± 7.32	11.8 (8.70, 13.0)
Liver and bile duct	5	4	9	11.3	46.0 ± 11.2	53.5 ± 14.1	4.96 (3.15, 9.34)
Breast		8	8	10.0	47.3 ± 7.31	53.0 ± 7.92	5.72 (2.64, 9.03)
Colorectal	2	4	6	7.5	47.8 ± 9.91	62.8 ± 7.80	13.0 (12.2, 14.4)
Trachea, lung, bronchus	5	1	6	7.5	57.0 ± 6.04	61.4 ± 6.73	4.67 (3.24, 6.23)
Other solid malignancies, unspecified	1	1	2	2.5	54.8 ± 5.59	58.7 ± 6.54	3.79 (3.12, 4.46)
Cervix		3	3	3.8	57.1 ± 2.77	61.5 ± 5.53	3.49 (1.89, 7.85)
Gallbladder	1	0	1	1.3	38.6	40.5	1.96
Kidney	3	0	3	3.8	55.0 ± 6.77	62.9 ± 4.29	9.36 (4.43, 9.94)
Thyroid	0	2	2	2.5	49.6 ± 23.8	53.1 ± 21.3	3.43 (1.64, 5.22)
Stomach	1	0	1	1.3	63.7	77.0	13.3
Ovary		1	1	1.3	60.9	63.3	2.43
Uterus, part unspecified		1	1	1.3	35.6	47.4	11.8
Total	38	42	80	100			
Hematologic							
NHL							
Monomorphic B cell	15	8	23	71.9	47.1 ± 10.2	57.2 ± 11.1	11.4 (4.11, 15.4)
Polymorphic	1	3	4	12.5	49.0 ± 12.7	54.6 ± 11.0	4.16 (4.11, 4.57)
Monomorphic T cell	2	0	2	6.3	37.3 ± 13.7	44.5 ± 19.6	4.84 (1.61, 12.7)
Leukemia, all types	1	1	2	6.3	54.1 ± 6.52	58.4 ± 6.23	4.36 (4.16, 4.57)
HL	1	0	1	3.1	52.3	60.1	7.78
Total	20	12	32	100			
Skin							
SCC	12	4	16	76.2	50.5 ± 7.31	63.0 ± 7.02	10.7 (7.10, 18.8)
BCC	4	1	5	23.8	52.4 ± 14.4	62.4 ± 8.73	7.62 (3.15, 15.3)
Total non-melanoma	16	5	21	100			

Abbreviations: BCC, basal cell carcinoma; HL, Hodgkin’s lymphoma; IQR, interquartile range; NHL, non-Hodgkin’s lymphoma; PTM, post-transplant malignancy; SCC, squamous cell carcinoma; SD, standard deviation.

Table 3 shows the SIRs adjusted for age, sex, and place of residence, comparing the KTRs to the Thai general population. The excess risk of *de novo* PTMs in KTRs was nearly fourfold compared with the general population. The top three cancers with significantly increased risk were consistent across genders. This risk was more pronounced for urothelial cancer, NHL, and non-melanoma skin cancer, all of which had more than a 20-fold excess risk. In particular, urothelial cancer showed the highest significant excess risk in women with a SIR of 114.7 (95% CI 66.8, 183.6). The risk of the most common cancers in the Thai general population, including lung, colorectal, breast, ovarian, and cervical cancers was comparable. The SIR for prostate cancer was significantly increased [SIR 8.11 (95% CI 3.71, 15.4)]. The SIR calculations considering only the first *de novo* PTM are shown in Supplementary Table S1.

**TABLE 3:** Standardized incidence ratios of different types of malignancies in kidney transplant patients by sex.

Cancer site	ICD-10	Male patients		Female patients		All	
		O/E	SIR (95% CI)	O/E	SIR (95% CI)	O/E	SIR (95% CI)
Prostate	C61	9/1.109145	8.11 (3.71, 15.4)*				
Breast	C50			8/5.166225	1.56 (0.67, 3.08)		
Ovary	C56			1/0.7406286	1.35 (0.36, 5.07)		
Cervix	C53			3/1.728333	1.73 (0.86, 4.91)		
Bronchus, lung, trachea	C33, C34	5/4.185616	1.20 (0.39, 2.79)	1/1.232264	0.81 (0.02, 4.52)	6/5.41788	1.11 (0.41, 2.41)
Stomach	C16	1/0.8211308	1.22 (0.03, 6.79)	0/0.4394963	0.0 (0.0, 6.82)	1/1.260627	0.79 (0.02, 4.42)
Liver and bile duct	C22, C24	5/6.909814	0.73 (0.24, 1.55)	4/1.381054	2.90 (0.79, 7.42)	9/8.290868	1.09 (0.50, 2.06)
Gallbladder	C23	1/0.0948561	10.5 (0.27, 58.7)	0/0.0948561	0 (0.0, 27.1)	1/0.2053207	4.87 (0.12, 27.1)
Colorectal	C18 - C20	2/3.178986	0.63 (0.08, 2.27)	4/1.370327	2.92 (0.80, 7.47)	6/4.549313	1.32 (0.48, 2.87)
Kidney	C64	3/0.2353302	12.7 (2.63, 37.3)*	0/0.0575237	0.0 (0.0, 52.1)	3/0.2928539	10.2 (2.11, 29.9)*
Urothelial	C65 - C67	11/0.6300718	17.5 (8.72, 31.2)*	17/0.1482415	114.7 (66.8, 183.6)*	28/0.6227172	36.0 (23.9, 52.0)*
Thyroid	C73	0/0.2594859	0 (0.0, 11.5)	2/0.5199386	3.85 (0.47, 13.9)	2/0.7794246	2.57 (0.31, 9.27)
Uterus, part unspecified	C55			1/0.0882458	11.3 (0.29, 63.1)		
Other solid malignancies: unspecified	O & U	1/1.03719	0.96 (0.02, 5.37)	1/0.3765939	2.66 (0.07, 14.8)	2/1.413784	1.42 (0.17, 5.11)
All solid malignancies		38/18.461626	2.06 (1.46, 2.83)*	42/13.343727	3.15 (2.27, 4.26)*	80/31.805353	2.52 (1.99, 3.13)*
NHL**	C82-85, C96	18/0.9139875	19.7 (11.7, 31.1)*	11/0.5157974	21.3 (10.6, 38.2)*	29/1.429785	20.3 (13.6, 29.1)*
HL	C81	1/0.0270835	36.9 (0.94, 205.7)	0/0.0121523	0 (0.0, 246.5)	1/0.0392358	25.5 (0.65, 142.0)
Leukemia	C91, C92-C94	1/0.3327671	3.01 (0.08, 16.7)	1/0.136444	7.33 (0.19, 40.8)	2/0.4692111	4.26 (0.52, 15.4)
All hematologic malignancies		20/1.2738381	15.7 (9.59, 24.2)*	12/0.6643937	18.1 (9.33, 31.6)*	32/1.9382318	16.5 (11.3, 23.3)*
Non-melanoma skin cancer***	C44	16/0.5000404	32.0 (18.3, 52.0)*	5/0.3501561	14.3 (4.64, 33.3)*	21/0.8501965	24.7 (15.3, 37.8)*
All solid and hematologic malignancies		58/19.735464	2.94 (2.23, 3.80)*	54/14.008121	3.86 (2.90, 5.03)*	112/33.743585	3.32 (2.73, 3.99)*
All cancers		74/20.235504	3.66 (2.87, 4.59)*	59/14.358277	3.76 (2.82, 4.91)*	133/34.593781	3.85 (3.22, 4.56)*

Notes: *Significant results at 95% confidence interval. ** Includes monomorphic B cells, and polymorphic, monomorphic T cells. *** Includes squamous cell carcinoma and basal cell carcinoma. The total risk time for the cohort was 16,495 person-years for standardized incidence ratio calculations.

Abbreviations: CI, confidence interval; E, expected; HL, Hodgkin’s lymphoma; ICD-10, international classification of diseases 10; NHL, non-Hodgkin’s lymphoma; O, observed; SIR, standardized incidence ratio.

Table 4 shows the SIRs for all PTMs stratified by age at transplant, duration of follow-up, and era of transplantation. The excess risk in KTRs compared to the general population by age at transplant was greatest in KTRs aged 45–64 years, and there was a comparable risk in KTRs transplanted at <20 years of age. By duration of follow-up, there was a decreasing trend over the duration of follow-up until the risk was comparable to the general population at 15 years or more of follow-up.

**TABLE 4:** Standardized incidence ratios for all malignancies stratified by age at transplantation, duration of follow-up, and year of transplantation.

Parameter	Person-years	O/E	SIR (95% CI)
Age at transplant, y			
<20	191.9535	1/0.0729803	13.7 (0.35, 76.3)
20–44	9,859.359	38/14.12602	2.69 (1.90, 3.69)
45–64	6,247.127	88/27.16186	3.24 (2.60, 3.99)
≥ 65	196.1123	6/1.928599	3.11 (1.14, 6.77)
Duration of follow-up, y			
<1	31.59206	6/0.069681	86.1 (31.6, 187)
1–5	2,100.715	39/4.575053	8.52 (6.06, 11.7)
5–10	4,465.733	39/11.0498	3.53 (2.51, 4.83)
10–15	3,839.713	24/10.8994	2.20 (1.41, 3.28)
15–19	3,067.138	14/8.118509	1.72 (0.94, 2.89)
≥20	2,989.662	11/8.577023	1.28 (0.64, 2.30)
Year of transplant			
Before 1989	191.2663	0/0.4592726	0
1989–1998	4,544.882	38/12.89968	2.95 (2.09, 4.04)
1999–2008	6,207.307	57/16.68373	3.42 (2.59, 4.43)
2009–2018	5,551.097	38/13.24678	2.87 (2.03, 3.94)

Abbreviations: CI, confidence interval; E, expected; O, observed; SIR, standardized incidence ratio.

Table 5 shows the incidence rates and SIRs for the top three most common PTMs in Thai KTRs, which were urothelial cancer, NHL, and non-melanoma skin cancer, stratified by age at transplant, duration of follow-up, and era of transplant. All three cancers showed no excess risk in KTRs aged ≥65 years at transplantation. For urothelial cancer, age at transplant between 20 and 64 years old showed significant excess risk with the greatest excess between 45–64 years old [SIR 28.4 (95% CI 17.1, 44.4)], and duration of follow-up showed the greatest significant excess risk between 1 and 5 years of follow-up. There was still a significant excess risk at ≥20 years of follow-up. NHL had the greatest excess risk by age at transplant in KTRs aged less than 20 years at transplant [SIR 362 (95% 9.15, 2,015)], and the excess risk decreased with increasing age at transplant. There was a significant excess risk of NHL at all follow-up periods, with the risk decreasing over time. For non-melanoma skin cancer, the greatest excess risk according to age at transplantation was between 20 and 44 years old [SIR 1,820 (95% CI 46.1, 10,139)], and there was excess risk up to >20 years of follow-up.

**TABLE 5:** Incidence rates per 100000 person-years at risk and standardized incidence ratios of selected cancers.

	Cancer type									
	Urothelial cancer			Non-Hodgkin’s lymphoma			Non-melanoma skin cancer			
	No. of events	Incidence rate per 100,000 person-years at risk (95% CI)	SIR (95% CI)	No. of events	Incidence rate per 100,000 person-years at risk (95% CI)	SIR (95% CI)	No. of events	Incidence rate per 100,000 person-years at risk (95% CI)	SIR (95% CI)	
Age at transplant										
<20	0		0	1	521 (73, 3,698)	362 (9.15, 2,015)*	0	0	0	
20–44	8	81 (41, 162)	18.7 (8.09, 36.9)*	11	112 (62, 201)	25.5 (12.8, 45.7)*	5	51 (21, 122)	1,820 (46.1, 10,139)*	
45–64	19	306 (195, 480)	28.4 (17.1, 44.4)*	16	256 (157, 418)	19.2 (11.0, 31.1)*	15	240 (145, 398)	28.5 (16.0, 47.0)*	
≥65	1	371 (52, 2,640)	15.3 (0.39, 85.4)	1	510 (72, 3,620)	17.4 (0.44, 96.8)	1	510 (72, 3,620)	22.3 (0.57, 124)	
Duration of follow-up, y										
<1	0		0	4	12,661 (4,752, 33,735)	1,903 (518, 4,872)*	1	3,165 (446, 22,471)	915 (23.2, 5,100)*	
1–5	8	381 (190, 761)	86.2 (37.2, 170)*	6	286 (128, 635)	42.5 (15.6, 92.4)*	2	95 (24, 381)		30.1 (3.65, 109)*
5–10	13	291 (169, 501)	51.8 (27.6, 88.6)*	4	89.6 (34, 239)	12.0 (3.27, 30.7)*	7	157 (75, 329)		39.5 (15.9, 81.5)*
10–15	2	52 (13.0, 208)	7.29 (0.88, 26.3)	8	208 (104, 417)	23.9 (10.3, 47.1)*	2	52 (13, 208)		10.5 (1.27, 38.0)*
15–19	2	65 (16, 261)	11.8 (1.43, 42.5)*	7	228 (109, 479)	28.5 (11.5, 58.8)*	5	163 (68, 392)		34.6 (11.2, 80.8)*
≥20	3	100 (32, 311)	13.8 (2.84, 40.3)*	0		0	4	134 (50, 356)		25.0 (6.81, 64.0)*
Year of transplant										
Before 1989	0		0	0		0	0			0
1989–1998	8	176 (88, 352)	25.4 (11.0, 50.0)*	6	132 (59, 294)	15.0 (5.50, 32.6)*	9	198 (103, 381)		38.9 (17.8, 73.8)*
1999–2008	14	226 (134, 381)	37.5 (20.5, 62.9)*	15	242 (146, 401)	29.9 (16.7, 49.3)*	6	97 (43, 215)		20.3 (7.46, 44.3)*
2009–2018	6	108 (49, 241)	20.7 (7.59, 45.0)*	8	144 (72, 288)	19.8 (8.56, 39.1)*	6	108 (49, 241)		29.0 (10.6, 63.1)*

Notes: *Significant results at 95% confidence interval.

Abbreviations: CI, confidence interval; E, expected; O, observed; SIR, standardized incidence ratio; Y, year.

Supplementary Figures S1–S3 show the cumulative incidence functions (CIF) of the three most common cancers adjusted for competing risks. After adjusting for all-cause death, graft failure, loss to follow-up, or administrative censoring, the odds of developing any of the three cancers were mostly low, at less than 5% over a period of nearly 20 years post-kidney transplantation.

## Discussion

We conducted the largest study of the excess risk of *de novo* PTMs in Thai KTRs at the highest volume transplant center in Thailand, accounting for a quarter of all historic kidney transplants in Thailand. The excess risk of *de novo* PTMs in our KTRs was approximately four times that of the general population after adjusting for age, sex, and region of residence, which is similar to the excess risk reported in other East Asian countries [2–4]. Furthermore, the top three cancers with excess risk among KTRs were urothelial cancer, NHL, and non-melanoma skin cancer. The subgroup of KTRs with the greatest excess risk of one type of PTM were female KTRs with urothelial cancer, who had a greater than 100-fold excess risk.

KTRs are susceptible to new viral infections or the reactivation of a latent one. The risk of oncogenic virus-related cancers, such as Kaposi’s sarcoma, NHL, and lip cancer, has been reported to be up to 10- to 100 times higher in KTRs than in the end-stage renal disease population [6, 7]. In the present study, urothelial cancer, NHL, and non-melanoma skin cancer all showed a more than 20-fold significant excess risk, and these have been associated with infection with BK virus or human papillomavirus (HPV), Epstein-Barr virus (EBV), and human papillomavirus infection, respectively [12–14]. Conversely, cervical cancer, associated with HPV infection, was not significant. Hepatitis B infection has been estimated to affect up to 6%–9% of the general Thai population born before the national hepatitis B vaccination program was rolled out in 1992 [15]. Unfortunately, the excess risk in KTRs of hepatocellular carcinoma associated with hepatitis B or C virus infection was uninterpretable in our study because it was coded together with bile duct cancer in some of our data sources.

In non-oncogenic virus-related cancers, susceptible KTRs with genetic risk factors or exposure to carcinogens may have preferential cancer cell transformation and growth due to immunosuppressive agents that affect DNA repair, immune surveillance, and pro- or anti-inflammatory cytokines [16, 17]. In the present study, we found just over a 10-fold excess risk for kidney cancer. Goh et al. reported that long dialysis vintage and native renal cysts predicted an increased risk of this cancer, which may be due to prolonged exposure to uremic toxin-induced oncogenic changes of epithelial cells [18]. With regard to prostate cancer, we are not aware of any pathophysiologic mechanism related to KTRs. The significance of the finding may be due to more opportunistic screening of prostate-specific antigen testing by urologists following up on KTR patients to find *de novo* PTMs than in the general population, or random sampling error.

With regard to urothelial cancer, we found that excess risk was observed in both sexes, but the excess risk was much more pronounced in female KTRs at more than 100-fold. Similar excess risks have been reported in population-based studies in Korea, Hong Kong, and particularly Taiwan, where the SIR for urothelial cancer was also greater than 100-fold [2–4]. In Europe, Australia, New Zealand, and the United States, although the absolute risk seems to be higher in male KTRs, the SIRs remain higher in female KTRs (SIRs 2.7–6.9), but they have been reported to be much higher in Asia [19, 20]. The reasons for this difference between the sexes remain to be elucidated. Of note, the majority of urothelial cancers in our KTRs were upper tract cancers, similar to the Taiwanese and Korean KTRs [21, 22]. The main risk factors contributing to this cancer in Taiwanese KTRs were exposure to herbal remedies containing Aristolochia and heavy metals in groundwater [22]. In the present study, we suspect that local Thai/Chinese herbal remedies may have been a contributing factor, although we did not collect data on such herbal remedies in this retrospective study. The hypothesis of a link with these herbal remedies is plausible because up to 45% of Thai patients with chronic kidney disease reported using local Thai/Chinese herbal remedies in the previous year [23]. Although the active ingredients of Thai/Chinese herbal remedies are not well documented, at least one herb, *Aristolochia acuminata,* known as “Krai-Krue,” has been identified by sampling polyherbal pills from pharmacies in Bangkok [24]. Furthermore, unregulated local Thai/Chinese herbal products may be contaminated with toxic heavy metals and analgesic agents, posing additional risks to users [25]. However, further study of these possible etiologic factors in Thai KTRs is needed to estimate the increased risk.

The excess risk of hematologic malignancies in KTRs is well-established. Epstein-Barr virus infection, along with the administration of T cell-depleting agents, can lead to the early development of post-transplant lymphoproliferative disorders (PTLD) [26]. In Asia, late-onset PTLD, defined as >1 year, was more common and was associated with an increased proportion of EBV-negative PTLD [27, 28]. Current KDIGO guidelines suggest EBV serologic testing before transplantation and monitoring for EBV reactivation in high-risk EBV mismatch recipients. Immunosuppression is suggested to be reduced if the EBV viral load increases during the follow-up [29]. Although no standard chemoprophylaxis regimen for EBV exists, a cohort study of 4,765 organ transplant recipients reported that no participant receiving a rituximab-containing induction regimen developed PTLD [30]. Reducing the dose of T cell-depleting agents was also found to reduce the risk of EBV reactivation, and showed a trend toward reducing the incidence of early PTLD without compromising graft survival over 2 years of follow-up [31].

The three most common skin cancers reported in KTRs are melanoma, non-melanoma, and Kaposi’s sarcoma, and this has been shown to be a significantly increased risk compared to the general population worldwide [32]. In the present study, *de novo* incident cases of non-melanoma skin cancer were observed while neither melanoma nor Kaposi’s sarcoma occurred. The risk of developing non-melanoma skin cancer can be reduced by educating patients about lifestyle modifications, such as smoking cessation and reducing exposure to ultraviolet light. In addition, in patients with a history of cancer, the physician may consider avoiding the prescription of azathioprine or the use of high-dose mycophenolate and may prefer to prescribe an mTOR inhibitor instead if the patient’s condition is appropriate [33, 34].

In general, the excess risk of PTMs rises in the first year post-transplantation, after which it falls over time. Highly elevated excess risk in the early post-transplantation period may not be truly *de novo* cancers, but rather cancers that developed prior to transplantation that were missed by preoperative screening. Furthermore, it is noteworthy that not all cancer types behave similarly. Krishnan et al. found that urinary tract cancer, melanoma, and post-transplant lymphoproliferative disease were the three most common incident cancers in the first year post-transplantation. In addition, incident lung and colorectal cancers were less common but were at a more advanced stage at the time of diagnosis [35].

Early detection of *de novo* PTM in KTRs through systematic cancer screening may provide a window of opportunity for effective early cancer treatment. Regarding the current cancer screening program in Thailand, the Thai Ministry of Public Health offers a 5-year PAP smear, bi-annual fecal occult blood, and opportunistic mammogram as population cancer screenings [11]. However, cancer screening among KTRs at a tertiary care center may be more intensive because specialists are likely to order screening tests that comply with international society recommendations [36]. This practice is deemed to be in line with the clinical practice guidelines of transplant organizations, which suggest that screening of KTRs should follow the provisions for systematic screening in the general population, with additional screening for other common cancers seen in KTRs, such as skin cancer [37]. Although this policy may seem to help detect common cancers, it may not cause survival benefits and may not be cost-effective due to the decreased life expectancy in KTRs, or it may inadvertently cause harm due to false-positive results, leading to over-investigation and psychological distress [38, 39].

The extremely high excess risk of urothelial cancer in KTRs in our study highlights the need for future action. Despite the aforementioned limited benefit of systematic screening programs in the general population, screening may be beneficial if the cancer incidence is high, the screened candidate has a high risk of cancer with a low risk of graft failure, and the screening tools are low-cost with high sensitivity [40]. From the results of the present study, we suggest that the special KTR subpopulation of women aged 45–65 years with an extremely high excess risk of urothelial cancer may be an initial candidate subgroup for systematic screening, especially in those who have been exposed to carcinogens, such as exposure to aromatic amines (e.g., benzidine and β-naphthylamine), high-dose cyclophosphamide, arsenic from contaminated drinking water, and HPV or BK viral infection [22, 41]. The high incidence of upper tract cancers may require urinalysis and cytology in combination with contrast-enhanced computed tomography of the kidneys, ureters, and bladder as the primary screening test. Abdominal ultrasound should not be used in this setting as it has low sensitivity for detecting early-stage disease [42]. Of note, if the patient has aristolochic nephropathy, the most effective way to improve the cancer outcome may be prophylactic bilateral nephrectomy, followed by annual surveillance cystoscopy and urine cytology [43, 44]. Due to the widespread use of unregulated Thai/Chinese medicinal herbs in the Thai population, we suggest that, if possible, a urothelial sample be obtained during the surgical procedure for a kidney transplant to test it for aristolochic acid-adducted DNA, which is a hallmark biomarker of aristolochic acid exposure [45]. This may identify very high-risk patients, allowing for appropriate management.

### Strengths and Limitations

There are strengths and limitations to our study. One strength is that this is the first study to demonstrate the excess *de novo* cancer risk among the Thai KTRs in a large sample size with a long period of follow-up for most of the KTRs. In addition, all pathologic diagnoses were confirmed by retrospective chart review, and the stratification of the excess incidence based on subnational regional risk in the Thai general population adjusted for different underlying risk factors in each subnational region. However, there are also limitations. One limitation is the use of the 2013–2015 Thai general population. We would have preferred to adjust our statistics for excess risk in KTRs compared to the Thai general population incidence by calendar year. However, there is a limitation as we have mentioned in the methodology section. The reasons for potential bias in our estimated standardized incidence ratio due to our choice of Thai general population comparison group can be divided into differences in age and sex distribution in the Thai population in mid-2014 and differences in cancer incidence between 2013 and 2015 independent of age and sex (e.g., changes in the prevalence of other cancer risk factors or national/subnational systematic cancer screening) compared with an ideal comparison group adjusting for age, sex, and cancer incidence by calendar year. The age structure of the Thai population has changed in recent years toward an aging society [46]. Due to the association of older age with higher cancer incidence in most PTMs, recent calendar year data with an older population may have higher cancer incidence, creating a bias in our estimates toward lower SIRs. The incidence of different types of cancer has changed at different rates. For example, cancers of the liver, lung, stomach, and Hodgkin’s lymphoma have had declining incidence trends, while cancers of the colon, breast, ovary, prostate, kidney, and non-Hodgkin’s lymphoma have had increasing trends. Hence, the expected directions of bias are upward bias in SIRs for the former group of cancers with decreasing trends and downward bias for those with increasing trends. Given the strong magnitudes of the strength of association at the lower bounds of the 95% CIs for cancers with known viral etiologies, which are known to be more likely in KTRs due to long-term maintenance immunosuppression, we suggest that there are truly significantly greater risks of these types of PTMs, regardless of bias due to choice of population for indirect standardization and other methods of adjusting for confounders [47]. We also acknowledge that the lack of standardized mortality ratios in our study may not allow for the interpretation of the data as to whether Thai KTRs are genuinely an appropriate, special, and very high-risk population that requires service provision for systematic screening for some cancers. Only cancers with significant excess risk, adjusted for confounders, compared to the Thai general population are attractive targets for systematic screening service provision. This issue should be investigated in future studies, but such studies may be challenging due to historical vital statistics data quality issues [9].

## Conclusion

The risk of *de novo* cancer increased after kidney transplantation in Thai adults, especially oncogenic virus-related cancers. The post-transplant malignancies with the greatest excess risk, such as urothelial cancer, warrant further investigation of risk factors to establish whether a systematic screening program is needed for any special, very high-risk subgroups of KTRs.

## Data Availability

The raw data supporting the conclusion of this article will be made available by the authors, without undue reservation.

## Supplementary Material

Supplementary Figure S1 Cumulative incidence functions (cif) of all urothelial cancers.

Supplementary Figure S2 Cumulative incidence functions (cif) of all non-Hodgkin’s lymphomas.

Supplementary Figure S3 Cumulative incidence functions (cif) of non-melanoma skin cancer.

Supplementary Table S1 Standardized incidence ratios of different types of first *de novo* malignancy in post-kidney transplant patients by sex.

## Abbreviations

BCC, Basal cell carcinoma; CI, Confident interval; CIF, Cumulative incidence functions; CNI, Calcineurin inhibitor; E, Expected; EBV, Epstein-Barr virus; HL, Hodgkin’s lymphoma; HPV, Human papillomavirus; KDIGO, Kidney Disease Improving Global Outcomes; KTR, Kidney transplant recipient; NCI, National Cancer Institute; NHL, Non-Hodgkin’s lymphoma; ICD-10, International classification of diseases 10; IQR, Interquartile range; O, Observed; PTM, Post-transplant malignancies; SIR, Standardized incidence ratio; SCC, Squamous cell carcinoma; SD, Standard deviation; Y, Year.

## References

[1] Ying T Shi B Kelly PJ Pilmore H Clayton PA Chadban SJ. Death After Kidney Transplantation: An Analysis by Era and Time Post-Transplant. J Am Soc Nephrol (2020) 31(12):2887–99. Epub 20200909. 10.1681/ASN.2020050566 32908001 PMC7790214

[2] Cheung CY Lam MF Chu KH Chow KM Tsang KY Yuen SK Malignancies After Kidney Transplantation: Hong Kong Renal Registry. Am J Transpl (2012) 12(11):3039–46. Epub 20120806. 10.1111/j.1600-6143.2012.04209.x 22883513

[3] Jeong S Lee HS Kong SG Kim DJ Lee S Park MJ Incidence of Malignancy and Related Mortality After Kidney Transplantation: A Nationwide, Population-Based Cohort Study in Korea. Sci Rep (2020) 10(1):21398. Epub 2020/12/10. 10.1038/s41598-020-78283-5 33293655 PMC7722878

[4] Yeh CC Khan A Muo CH Yang HR Li PC Chang CH De Novo Malignancy After Heart, Kidney, and Liver Transplant: A Nationwide Study in Taiwan. Exp Clin Transpl (2020) 18(2):224–33. Epub 20200304. 10.6002/ect.2019.0210 32133940

[5] Benoni H Eloranta S Dahle DO Svensson MHS Nordin A Carstens J Relative and Absolute Cancer Risks Among Nordic Kidney Transplant Recipients-A Population-Based Study. Transpl Int (2020) 33(12):1700–10. Epub 20200925. 10.1111/tri.13734 32896035 PMC7756726

[6] Vajdic CM McDonald SP McCredie MR van Leeuwen MT Stewart JH Law M Cancer Incidence Before and After Kidney Transplantation. JAMA (2006) 296(23):2823–31. 10.1001/jama.296.23.2823 17179459

[7] Yanik EL Clarke CA Snyder JJ Pfeiffer RM Engels EA. Variation in Cancer Incidence Among Patients With Esrd During Kidney Function and Nonfunction Intervals. J Am Soc Nephrol (2016) 27(5):1495–504. Epub 20151112. 10.1681/ASN.2015040373 26563384 PMC4849829

[8] Sprangers B Nair V Launay-Vacher V Riella LV Jhaveri KD. Risk Factors Associated With Post-Kidney Transplant Malignancies: An Article From the Cancer-Kidney International Network. Clin Kidney J (2018) 11(3):315–29. Epub 2018/06/27. 10.1093/ckj/sfx122 29942495 PMC6007332

[9] Sirirungreung A Buasom R Jiraphongsa C Sangrajrang S. Data Reliability and Coding Completeness of Cancer Registry Information Using Reabstracting Method in the National Cancer Institute: Thailand, 2012 to 2014. J Glob Oncol (2018) 4:1–9. 10.1200/JGO.17.00147 PMC6223438 30241269

[10] Insamran WPA Supattagron P Chiawiriyabunya I Namthaisong K Wongsena K Puttawibul P Cancer in Thailand Vol.Ix, 2013-2015 (2018).

[11] Insamran W Sangrajrang S. National Cancer Control Program of Thailand. Asian Pac J Cancer Prev (2020) 21(3):577–82. Epub 20200301. 10.31557/APJCP.2020.21.3.577 32212781 PMC7437310

[12] Cantalupo PG Katz JP Pipas JM. Viral Sequences in Human Cancer. Virology (2018) 513:208–16. Epub 20171105. 10.1016/j.virol.2017.10.017 29107929 PMC5828528

[13] Manole B Damian C Giusca SE Caruntu ID Porumb-Andrese E Lunca C The Influence of Oncogenic Viruses in Renal Carcinogenesis: Pros and Cons. Pathogens (2022) 11(7):757. Epub 20220702. 10.3390/pathogens11070757 35890003 PMC9319782

[14] Opelz G Daniel V Naujokat C Dohler B. Epidemiology of Pretransplant Ebv and Cmv Serostatus in Relation to Posttransplant Non-Hodgkin Lymphoma. Transplantation (2009) 88(8):962–7. 10.1097/TP.0b013e3181b9692d 19855238

[15] Posuwan N Wanlapakorn N Sintusek P Wasitthankasem R Poovorawan K Vongpunsawad S Towards the Elimination of Viral Hepatitis in Thailand by the Year 2030. J Virus Erad (2020) 6(3):100003. Epub 20200627. 10.1016/j.jve.2020.100003 33251021 PMC7646674

[16] Herman M Weinstein T Korzets A Chagnac A Ori Y Zevin D Effect of Cyclosporin a on DNA Repair and Cancer Incidence in Kidney Transplant Recipients. J Lab Clin Med (2001) 137(1):14–20. 10.1067/mlc.2001.111469 11150019

[17] Morteau O Blundell S Chakera A Bennett S Christou CM Mason PD Renal Transplant Immunosuppression Impairs Natural Killer Cell Function *In Vitro* and *In Vivo* . PLoS One (2010) 5(10):e13294. Epub 2010/10/23. 10.1371/journal.pone.0013294 20967261 PMC2953494

[18] Goh A Vathsala A. Native Renal Cysts and Dialysis Duration Are Risk Factors for Renal Cell Carcinoma in Renal Transplant Recipients. Am J Transpl (2011) 11(1):86–92. Epub 20101025. 10.1111/j.1600-6143.2010.03303.x 20973916

[19] Stewart JH Buccianti G Agodoa L Gellert R McCredie MR Lowenfels AB Cancers of the Kidney and Urinary Tract in Patients on Dialysis for End-Stage Renal Disease: Analysis of Data From the United States, Europe, and Australia and New Zealand. J Am Soc Nephrol (2003) 14(1):197–207. Epub 2002/12/31. 10.1097/01.asn.0000039608.81046.81 12506152

[20] Kasiske BL Snyder JJ Gilbertson DT Wang C. Cancer After Kidney Transplantation in the United States. Am J Transpl (2004) 4(6):905–13. 10.1111/j.1600-6143.2004.00450.x 15147424

[21] Yu J Lee CU Kang M Jeon HG Jeong BC Seo SI Incidences and Oncological Outcomes of Urothelial Carcinoma in Kidney Transplant Recipients. Cancer Manag Res (2019) 11:157–66. Epub 2019/01/15. 10.2147/CMAR.S185796 30636892 PMC6307682

[22] Wu MJ Lian JD Yang CR Cheng CH Chen CH Lee WC High Cumulative Incidence of Urinary Tract Transitional Cell Carcinoma After Kidney Transplantation in Taiwan. Am J Kidney Dis (2004) 43(6):1091–7. Epub 2004/05/29. 10.1053/j.ajkd.2004.03.016 15168390

[23] Tangkiatkumjai M Boardman H Praditpornsilpa K Walker DM. Prevalence of Herbal and Dietary Supplement Usage in Thai Outpatients With Chronic Kidney Disease: A Cross-Sectional Survey. BMC Complement Altern Med (2013) 13:153. Epub 2013/07/03. 10.1186/1472-6882-13-153 23815983 PMC3750602

[24] Thongkhao K Tungphatthong C Sukrong S. A Pcr-Lateral Flow Immunochromatographic Assay (Pcr-Lfa) for Detecting Aristolochia Species, the Plants Responsible for Aristolochic Acid Nephropathy. Sci Rep (2022) 12(1):12188. Epub 20220716. 10.1038/s41598-022-16528-1 35842504 PMC9288547

[25] Ernst E. Toxic Heavy Metals and Undeclared Drugs in Asian Herbal Medicines. Trends Pharmacol Sci (2002) 23(3):136–9. 10.1016/S0165-6147(00)01972-6 11879681

[26] Ali H Soliman K Daoud A Elsayed I Fulop T Sharma A Relationship Between Rabbit Anti-Thymocyte Globulin and Development of Ptld and its Aggressive Form in Renal Transplant Population. Ren Fail (2020) 42(1):489–94. 10.1080/0886022X.2020.1759636 32423337 PMC7301714

[27] Petrara MR Giunco S Serraino D Dolcetti R De Rossi A. Post-Transplant Lymphoproliferative Disorders: From Epidemiology to Pathogenesis-Driven Treatment. Cancer Lett (2015) 369(1):37–44. Epub 20150813. 10.1016/j.canlet.2015.08.007 26279520

[28] Cheung CY Ma MKM Chau KF Chak WL Tang SCW. Posttransplant Lymphoproliferative Disorders in Kidney Transplant Recipients: A Retrospective Cohort Analysis Over Two Decades in Hong Kong. Oncotarget (2017) 8(57):96903–12. Epub 20170630. 10.18632/oncotarget.18890 29228580 PMC5722532

[29] Kidney Disease: Improving Global Outcomes (KDIGO) Transplant Work Group. Kdigo Clinical Practice Guideline for the Care of Kidney Transplant Recipients. Am J Transpl (2009) 9(3):S1–155. Epub 2009/10/23. 10.1111/j.1600-6143.2009.02834.x 19845597

[30] Walti LN Mugglin C Sidler D Mombelli M Manuel O Hirsch HH Association of Antiviral Prophylaxis and Rituximab Use With Posttransplant Lymphoproliferative Disorders (PTLDs): A Nationwide Cohort Study. Am J Transpl (2021) 21(7):2532–42. Epub 20201222. 10.1111/ajt.16423 PMC8359347 33289340

[31] Ashoor IF Beyl RA Gupta C Jain A Kiessling SG Moudgil A Low-Dose Antithymocyte Globulin Has No Disadvantages to Standard Higher Dose in Pediatric Kidney Transplant Recipients: Report From the Pediatric Nephrology Research Consortium. Kidney Int Rep (2021) 6(4):995–1002. Epub 20210117. 10.1016/j.ekir.2021.01.007 33912749 PMC8071617

[32] Au E Wong G Chapman JR. Cancer in Kidney Transplant Recipients. Nat Rev Nephrol (2018) 14(8):508–20. 10.1038/s41581-018-0022-6 29802400

[33] Shao EX Betz-Stablein B Marquat L Campbell S Isbel N Green AC Higher Mycophenolate Dosage Is Associated With an Increased Risk of Squamous Cell Carcinoma in Kidney Transplant Recipients. Transpl Immunol (2022) 75:101698. Epub 20220819. 10.1016/j.trim.2022.101698 35988897

[34] Thet Z Lam AK Ranganathan D Aung SY Han T Khoo TK. Reducing Non-Melanoma Skin Cancer Risk in Renal Transplant Recipients. Nephrology (Carlton) (2021) 26(11):907–19. Epub 20210728. 10.1111/nep.13939 34240786

[35] Krishnan A Wong G Teixeira-Pinto A Lim WH. Incidence and Outcomes of Early Cancers After Kidney Transplantation. Transpl Int (2022) 35:10024. Epub 20220503. 10.3389/ti.2022.10024 35592449 PMC9110645

[36] Pausawasdi N Tongpong P Geeratragool T Charatcharoenwitthaya P. An Assessment of Physicians' Recommendations for Colorectal Cancer Screening and International Guidelines Awareness and Adherence: Results From a Thai National Survey. Front Med (Lausanne) (2022) 9:847361. Epub 20220429. 10.3389/fmed.2022.847361 35572969 PMC9100397

[37] Acuna SA Huang JW Scott AL Micic S Daly C Brezden-Masley C Cancer Screening Recommendations for Solid Organ Transplant Recipients: A Systematic Review of Clinical Practice Guidelines. Am J Transpl (2017) 17(1):103–14. Epub 20160830. 10.1111/ajt.13978 27575845

[38] Boenink R Astley ME Huijben JA Stel VS Kerschbaum J Ots-Rosenberg M The Era Registry Annual Report 2019: Summary and Age Comparisons. Clin Kidney J (2022) 15(3):452–72. Epub 20211215. 10.1093/ckj/sfab273 35211303 PMC8862051

[39] Wong G Chapman JR Craig JC. Cancer Screening in Renal Transplant Recipients: What Is the Evidence?. Clin J Am Soc Nephrol (2008) 3(2):S87–S100. 10.2215/CJN.03320807 18309007 PMC3152279

[40] Wong G Howard K Webster AC Chapman JR Craig JC. Screening for Renal Cancer in Recipients of Kidney Transplants. Nephrol Dial Transpl (2011) 26(5):1729–39. Epub 2010/10/22. 10.1093/ndt/gfq627 20961889

[41] Pradere B Schuettfort V Mori K Quhal F Aydh A Sari Motlagh R. Management of De-Novo Urothelial Carcinoma in Transplanted Patients. Curr Opin Urol (2020) 30(3):467–74. Epub 2020/04/03. 10.1097/MOU.0000000000000749 32235285

[42] Kliem V Thon W Krautzig S Kolditz M Behrend M Pichlmayr R High Mortality From Urothelial Carcinoma Despite Regular Tumor Screening in Patients With Analgesic Nephropathy After Renal Transplantation. Transpl Int (1996) 9(3):231–5. 10.1007/BF00335391 8723192

[43] Lemy A Wissing KM Rorive S Zlotta A Roumeguere T Muniz Martinez MC Late Onset of Bladder Urothelial Carcinoma After Kidney Transplantation for End-Stage Aristolochic Acid Nephropathy: A Case Series With 15-Year Follow-Up. Am J Kidney Dis (2008) 51(3):471–7. 10.1053/j.ajkd.2007.11.015 18295063

[44] Kanaan N Hassoun Z Raggi C Jadoul M Mourad M De Meyer M Long-Term Outcome of Kidney Recipients Transplanted for Aristolochic Acid Nephropathy. Transplantation (2016) 100(2):416–21. 10.1097/TP.0000000000000941 26457602

[45] Jelakovic B Karanovic S Vukovic-Lela I Miller F Edwards KL Nikolic J Aristolactam-DNA Adducts Are a Biomarker of Environmental Exposure to Aristolochic Acid. Kidney Int (2012) 81(6):559–67. Epub 20111109. 10.1038/ki.2011.371 22071594 PMC3560912

[46] United Nation Department of Economic and Social Affairs Population Division. World Population Prospective 2022 (2022). Available From: https://population.un.org/wpp/Graphs/DemographicProfiles/Pyramid/764 (Accessed February 1, 2023).

[47] Blair A Stewart P Lubin JH Forastiere F. Methodological Issues Regarding Confounding and Exposure Misclassification in Epidemiological Studies of Occupational Exposures. Am J Ind Med (2007) 50(3):199–207. 10.1002/ajim.20281 17096363

