# Supplementary figures and images for: Incidence of De Novo Post-Transplant Malignancies in Thai Adult Kidney Transplant Recipients: A Single-Center, Population-Controlled, Retrospective Cohort Study at the Highest Volume Kidney Transplant Center in Thailand
Source: Transpl Int. 2024 Feb 26;37:11614. doi: 10.3389/ti.2024.11614 (PMC10926888; doi:10.3389/ti.2024.11614)

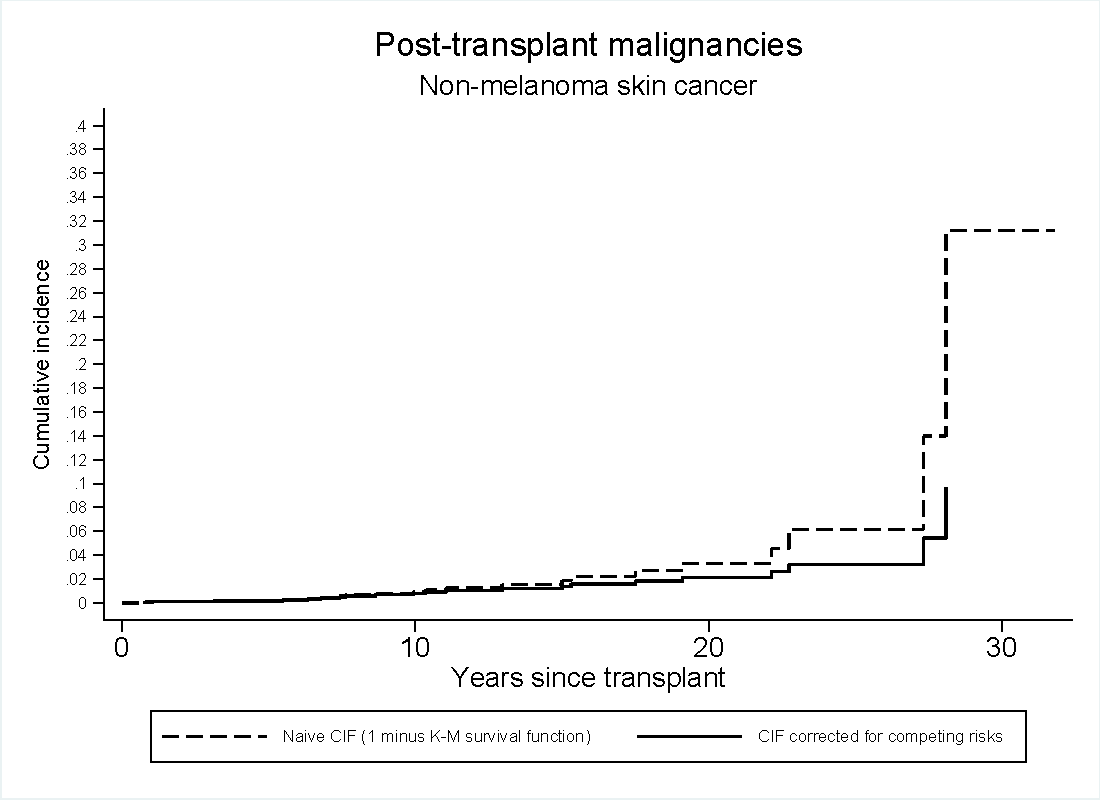

Supplement: Supplementary file 1 [file Image3.TIFF]

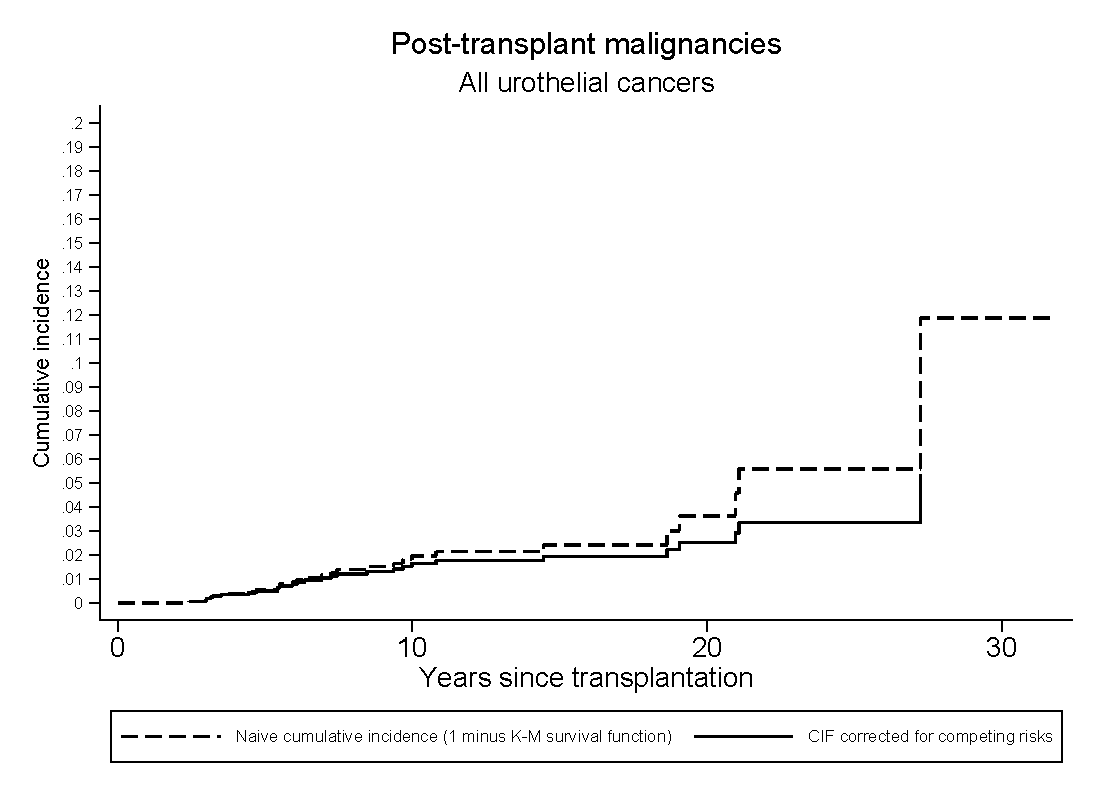

Supplement: Supplementary file 2 [file Image1.TIFF]

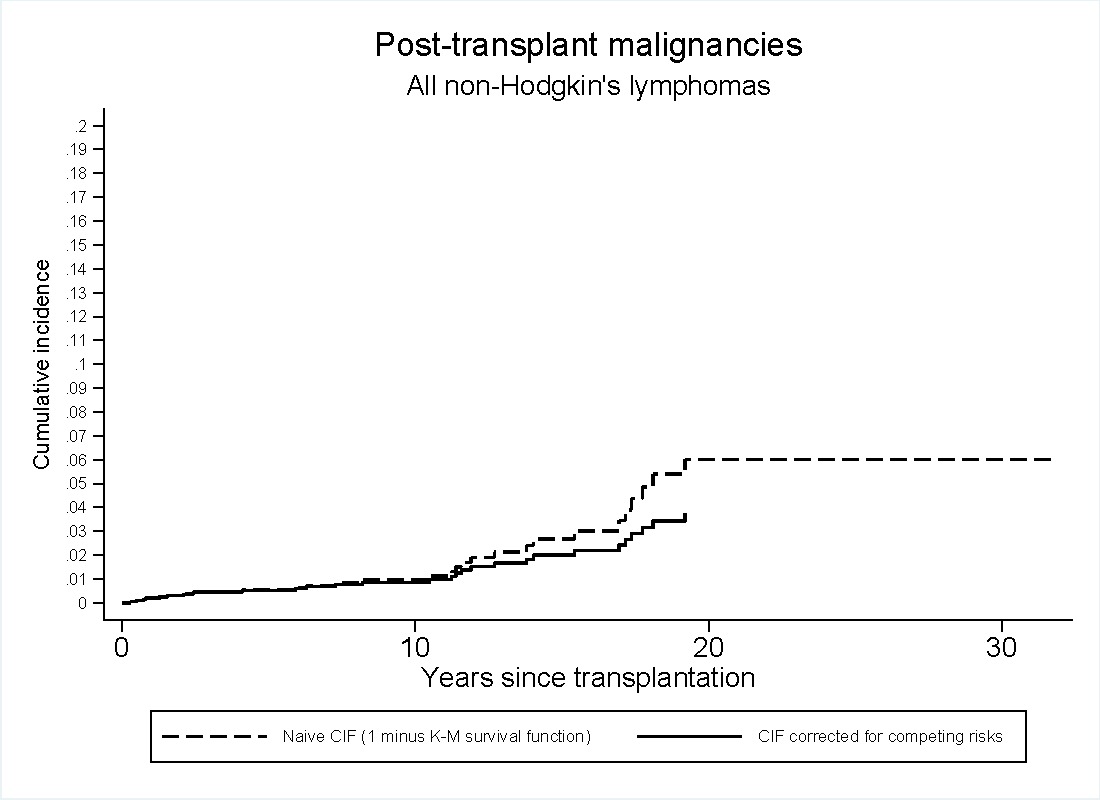

Supplement: Supplementary file 4 [file Image2.TIFF]
