# Supplementary material for: Incidence of De Novo Post-Transplant Malignancies in Thai Adult Kidney Transplant Recipients: A Single-Center, Population-Controlled, Retrospective Cohort Study at the Highest Volume Kidney Transplant Center in Thailand
Source: Transpl Int. 2024 Feb 26;37:11614. doi: 10.3389/ti.2024.11614 (PMC10926888; doi:10.3389/ti.2024.11614)
Supplement: Supplementary file 3 [file Table1.docx]

**Supplement Table1.** Standardized incidence ratios of different types of first incident malignancy during the follow-up in post-kidney transplant patients by sex

| **Site of cancer** | **ICD-10** | **Male** | |  | **Female** | |  | **All** | |
| --- | --- | --- | --- | --- | --- | --- | --- | --- | --- |
|  |  | **O/E** | **SIR (95%CI)** |  | **O/E** | **SIR (95%CI)** |  | **O/E** | **SIR (95%CI)** |
| **Prostate** | C61 | 8/0.8140512 | 9.82 (4.24, 19.4)* |  |  |  |  |  |  |
| **Breast** | C50 |  |  |  | 8/5.166341 | 1.55 (0.67, 3.05) |  |  |  |
| **Ovary** | C56 |  |  |  | 1/0.7406277 | 1.35 (0.03, 7.52) |  |  |  |
| **Cervix** | C53 |  |  |  | 3/1.728323 | 1.74 (0.36, 5.07) |  |  |  |
| **Bronchus, lung, trachea** | C33, C34 | 5/4.185637 | 1.20 (0.39, 2.79) |  | 1/1.232255 | 0.81 (0.02, 4.52) |  | 6/5.417892 | 1.11 (0.41, 2.41) |
| **Gastric** | C16 | 1/0.8211307 | 1.22 (0.03, 6.79) |  | 0/0.4394959 | 0.0 (0.0, 6.82) |  | 1/1.2606266 | 0.79 (0.02, 4.42) |
| **Liver and bile duct** | C22, C24 | 5/6.909789 | 0.72 (0.24, 1.69) |  | 2/1.381041 | 1.45 (0.18, 5.23) |  | 7/8.29083 | 0.84 (0.34, 1.74) |
| **Gallbladder** | C23 | 1/0.0948556 | 10.5 (0.27, 58.7) |  | 0/0.0948545 | 0 (0.0, 31.6) |  | 1/0.1897101 | 5.27 (0.13, 29.4) |
| **Colorectal** | C18 - C20 | 1/3.178974 | 0.32 (0.008, 1.75) |  | 4/1.370300 | 2.92 (0.80, 7.47) |  | 5/4.549274 | 1.10 (0.36, 2.57) |
| **Kidney** | C64 | 3/0.2353287 | 12.7 (2.63, 37.3)* |  | 0/0.0575222 | 0.0 (0.0, 52.1) |  | 3/0.2928509 | 10.2 (2.11, 29.9)* |
| **Urothelial** | C65 - C67 | 10/0.6300641 | 15.9 (7.61, 29.2)* |  | 16/0.1482371 | 107.9 (61.7, 175.3)* |  | 26/0.6227172 | 33.4 (21.8, 48.9)* |
| **Thyroid** | C73 | 0/0.2594849 | 0 (0.0, 11.5) |  | 2/0.5199375 | 3.85 (0.47, 13.9) |  | 2/0.7794224 | 2.57 (0.31, 9.27) |
| **Uterus, part unspecified** | C55 |  |  |  | 1/0.0882387 | 11.3 (0.29, 63.1) |  |  |  |
| **Other solid malignancies: unspecified** | O & U | 1/1.03675 | 0.97 (0.02, 5.37) |  | 1/0.3765804 | 2.66 (0.07, 14.8) |  | 2/1.4133304 | 1.42 (0.17, 5.11) |
| **All solid malignancies** |  | 34/18.166065 | 1.87 (1.30, 2.62)* |  | 39/13.343754 | 2.92 (2.08, 4.00)* |  | 73/31.509819 | 2.32 (1.81, 2.91)* |
| **NHL**** | C82-85, C96 | 18/0.9139861 | 19.7 (11.7, 31.1)* |  | 11/0.5157899 | 21.3 (10.6, 38.2)* |  | 29/1.429776 | 20.3 (13.6, 29.1)* |
| **HL** | C81 | 1/0.0270801 | 36.9 (0.94, 205.7) |  | 0/0.0121485 | 0 (0.0, 246.6) |  | 1/0.0392286 | 25.5 (0.65, 142.0) |
| **Leukemia** | C91, C92-C94 | 1/0.3327589 | 3.01 (0.08, 16.7) |  | 1/0.136372 | 7.33 (0.19, 40.9) |  | 2/0.4691309 | 4.26 (0.52, 15.4) |
| **All hematologic malignancies** |  | 20/1.2738381 | 15.7 (9.59, 24.2)* |  | 12/0.6643104 | 18.1 (9.33, 31.6)* |  | 32/1.9381485 | 16.5 (11.3, 23.3)* |
| **Non-melanoma skin cancer***** | C44 | 15/0.5000348 | 30.0 (16.8, 49.5)* |  | 5/0.3501404 | 14.3 (4.64, 33.3)* |  | 20/0.8501752 | 23.5 (14.4, 36.3)* |
| **All solid and hematologic malignancies** |  | 54/19.439903 | 2.78 (2.10, 3.62)* |  | 51/14.008064 | 3.64 (2.71, 4.79)* |  | 105/33.447967 | 3.14 (2.57, 3.80)* |
| **All cancers** |  | 69/19.939938 | 3.46 (2.69, 4.38)* |  | 56/14.358204 | 3.90 (2.95, 5.07)* |  | 125/34.298142 | 3.65 (3.03, 4.34)* |

Notes:- *Significant results by 95% confidence interval. ** Includes monomorphic B cell, polymorphic, monomorphic T cell. *** Includes squamous cell carcinoma and basal cell carcinoma. The total risk time for the cohort was 16,382 person-years for standardized incidence ratio calculations for only first incident *de novo* post-transplant malignancies.

Abbreviations: CI, confidence interval; E, expected; HL, Hodgkin lymphoma; ICD-10, international classification of diseases 10; NHL, non-Hodgkin lymphoma; O, observed; SIR, standardized incidence ratio
